# Total Body Irradiation in Haematopoietic Stem Cell Transplantation for Paediatric Acute Lymphoblastic Leukaemia: Review of the Literature and Future Directions

**DOI:** 10.3389/fped.2021.774348

**Published:** 2021-12-03

**Authors:** Bianca A. W. Hoeben, Jeffrey Y. C. Wong, Lotte S. Fog, Christoph Losert, Andrea R. Filippi, Søren M. Bentzen, Adriana Balduzzi, Lena Specht

**Affiliations:** ^1^Department of Radiation Oncology, University Medical Center Utrecht, Utrecht, Netherlands; ^2^Princess Máxima Center for Pediatric Oncology, Utrecht, Netherlands; ^3^Department of Radiation Oncology, City of Hope National Medical Center and Beckman Research Institute, Duarte, CA, United States; ^4^Alfred Health Radiation Oncology, The Alfred Hospital, Melbourne, VIC, Australia; ^5^Department of Radiation Oncology, University Hospital, Ludwig Maximilian University of Munich, Munich, Germany; ^6^Department of Radiation Oncology, Fondazione IRCCS Policlinico San Matteo and University of Pavia, Pavia, Italy; ^7^Division of Biostatistics and Bioinformatics, Department of Epidemiology and Public Health, University of Maryland School of Medicine, Baltimore, MD, United States; ^8^Stem Cell Transplantation Unit, Clinica Paediatrica Università degli Studi di Milano Bicocca, Monza, Italy; ^9^Department of Oncology, Rigshospitalet, University of Copenhagen, Copenhagen, Denmark

**Keywords:** haematopoietic stem cell transplantation (HSCT), total body irradiation (TBI), total marrow irradiation (TMI), total lymph node irradiation (TLI), acute lymphoblastic leukaemia (ALL), total marrow and lymphatic irradiation, paediatric

## Abstract

Total body irradiation (TBI) has been a pivotal component of the conditioning regimen for allogeneic myeloablative haematopoietic stem cell transplantation (HSCT) in very-high-risk acute lymphoblastic leukaemia (ALL) for decades, especially in children and young adults. The myeloablative conditioning regimen has two aims: (1) to eradicate leukaemic cells, and (2) to prevent rejection of the graft through suppression of the recipient's immune system. Radiotherapy has the advantage of achieving an adequate dose effect in sanctuary sites and in areas with poor blood supply. However, radiotherapy is subject to radiobiological trade-offs between ALL cell destruction, immune and haematopoietic stem cell survival, and various adverse effects in normal tissue. To diminish toxicity, a shift from single-fraction to fractionated TBI has taken place. However, HSCT and TBI are still associated with multiple late sequelae, leaving room for improvement. This review discusses the past developments of TBI and considerations for dose, fractionation and dose-rate, as well as issues regarding TBI setup performance, limitations and possibilities for improvement. TBI is typically delivered using conventional irradiation techniques and centres have locally developed heterogeneous treatment methods and ways to achieve reduced doses in several organs. There are, however, limitations in options to shield organs at risk without compromising the anti-leukaemic and immunosuppressive effects of conventional TBI. Technological improvements in radiotherapy planning and delivery with highly conformal TBI or total marrow irradiation (TMI), and total marrow and lymphoid irradiation (TMLI) have opened the way to investigate the potential reduction of radiotherapy-related toxicities without jeopardising efficacy. The demonstration of the superiority of TBI compared with chemotherapy-only conditioning regimens for event-free and overall survival in the randomised For Omitting Radiation Under Majority age (FORUM) trial in children with high-risk ALL makes exploration of the optimal use of TBI delivery mandatory. Standardisation and comprehensive reporting of conventional TBI techniques as well as cooperation between radiotherapy centres may help to increase the ratio between treatment outcomes and toxicity, and future studies must determine potential added benefit of innovative conformal techniques to ultimately improve quality of life for paediatric ALL patients receiving TBI-conditioned HSCT.

## Introduction

Since the 1970s total body irradiation (TBI) is considered to be a cornerstone of myeloablative conditioning for haematopoietic stem cell transplantation (HSCT) in children. It has been used in combination with chemotherapy as conditioning regimen both in autologous and allogeneic HSCT for malignant and non-malignant diseases (1). However, it gradually became clear that HSCT survivors suffered from various late adverse effects, many of which related to TBI (2–6). As HSCT strategies improved and evolved over time, and reduction of late sequelae was warranted, chemotherapy-only conditioning schedules (chemoconditioning) became the mainstay for most indications; the use of myeloablative TBI was limited mainly to patients with high-risk haematologic malignancies in the allogeneic setting (7–10). For most paediatric acute myeloblastic leukaemia (AML) HSCT indications, chemoconditioning gained preference over TBI-based conditioning (11–14). In children with very-high-risk acute lymphoblastic leukaemia (ALL), studies consistently showed superior survival outcomes of TBI-based conditioning (15–19).

**Figure 1:**
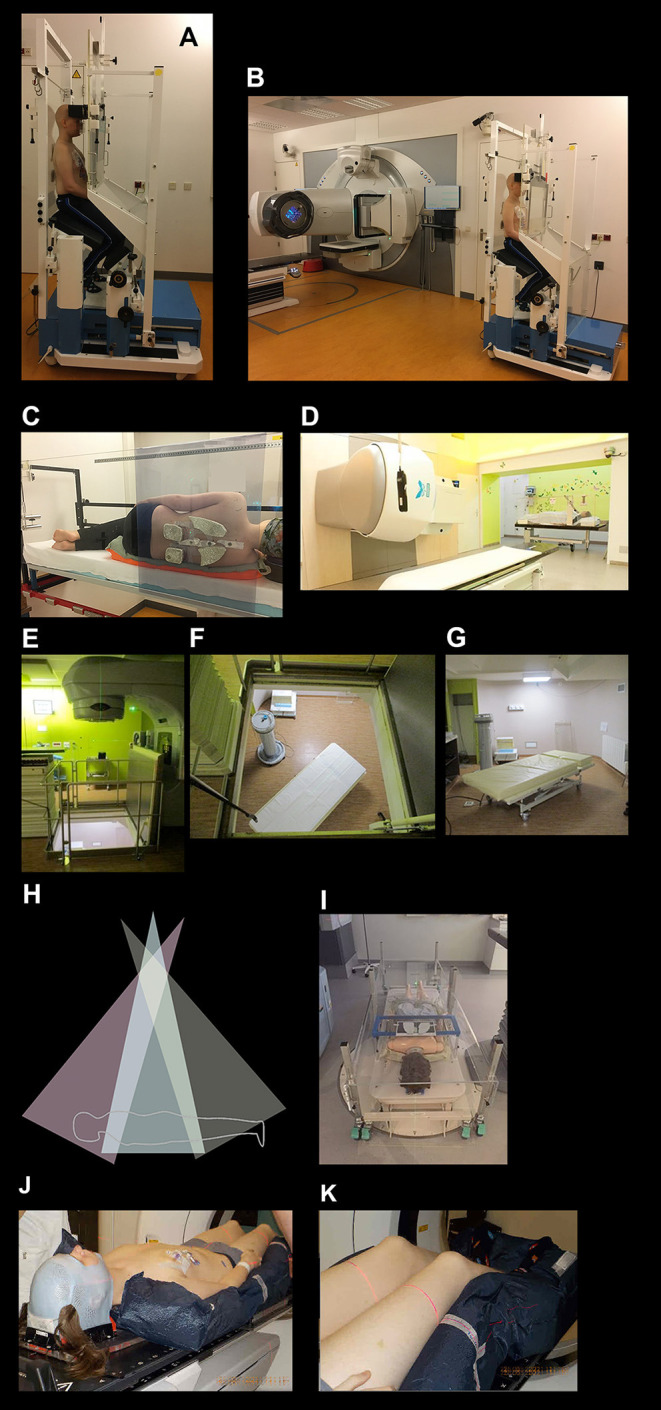
Total body irradiation setup examples. **(A,B)** A patient in an institution-developed TBI “chair” setup for opposed anterior/posterior (AP-PA) dose delivery with acrylic beam spoilers in front of and behind the patient; the chair is rotated 180° halfway through each fraction; shielding of lungs, kidneys, and lenses is performed with individually moulded cerrobend blocks. **(C)** A patient in an institution-developed TBI “bed” setup for AP-PA dose delivery in the lateral decubitus position, with beam spoilers; the patient is rotated 180° halfway through each fraction; shielding of lungs, kidneys, and lenses is performed with individually moulded cerrobend blocks. **(D)** A patient in an institution-developed TBI “bed” setup for lateral dose delivery in the supine position, with beam spoilers; the bed is rotated 180° halfway through each fraction and there is shielding of lungs. **(E–G)** An institution-developed TBI “bed” setup for AP-PA dose delivery where the linear accelerator gantry is positioned one floor above the patient, and the patient is rotated from the supine to prone position halfway through a fraction. **(H,I)** A sweeping-beam TBI “bed” setup for AP-PA dose delivery where the linear accelerator gantry is positioned ±2 m above the patient and sweeps stepwise in an arc over the entire body, delivering the dose in multiple static (up to 20) positions, thereby increasing dose homogeneity; the patient is rotated from the supine to prone position halfway through a fraction; beam spoilers cover the patient, with individually moulded lung blocks placed below the spoiler. **(J,K)** A patient in a highly conformal isocentric technique treatment position (e.g., VMAT TBI, TomoTherapy TBI, TMI, or TMLI) lying supine in a body-length vacuum bag and open head mask for secure positioning during treatment; as the gantry rotates around sequential isocentres in the body and table translations take place. TMI, total marrow irradiation; TMLI, total marrow and lymphoid irradiation; VMAT, volumetric-modulated arc therapy. Images **(E–G)** courtesy of S. Supiot, Institut de Cancérologie de l'Ouest, Nantes St. Herblain, France. Images **(H,I)** courtesy of L. Sim, Radiation Oncology Princess Alexandra Raymond Terrace, Brisbane, Queensland, Australia.

The aspiration to reduce acute and long-term effects after HSCT—especially in developing children—has motivated radiation oncologists to seek out improvements in TBI performance. For many years, myeloablative TBI was mostly given as a single fraction of up to 10 Gy combined with cyclophosphamide (20, 21). Gradually, studies showed decreased toxicities and equal or improved survival with fractionated TBI (22–24), and this has become the standard. However, institutions have developed site-specific TBI setups and techniques, making practises heterogeneous (25–28). With technological advances, general radiation treatments have evolved into highly conformal intensity-modulated techniques delivering high doses to treatment volumes while increasingly sparing the surrounding tissues. For TBI, however, most centres still use two-dimensional (2D) conventional techniques with opposing beams that capture the entire body while shielding certain organs at risk (OAR) (27, 28) (Figure 1). This technique tends to deliver heterogeneous doses throughout the body while shielding also blocks bone marrow compartments. Several centres have introduced highly conformal techniques that offer better dose homogeneity while allowing more options to spare OAR, albeit with higher dose rates than classical setups (29–31). More targeted radiotherapy strategies such as total marrow irradiation (TMI), total lymphoid irradiation (TLI), and total marrow and lymphoid irradiation (TMLI) allow dose escalation to the bone marrow and/or lymphoid volumes of high-risk ALL patients while reducing doses in the remainder of the body. Clinical studies to establish the role of TMLI in HSCT-conditioning are ongoing (32).

Since the superiority of including TBI in conditioning regimens prior to HSCT for very-high-risk ALL paediatric patients has been reinforced by the results of the For Omitting Radiation Under Majority age (FORUM) trial (19), it is timely to review TBI application and rationale for these patients and to gauge future directions.

## Studies of TBI-Based Conditioning for all

TBI has been the most frequently applied myeloablative conditioning for HSCT in patients with ALL. Most centres now avoid TBI in children below the age of 3 years because of increased side effects especially on the young developing brain. Prior to the FORUM trial, there was remaining debate over whether non-inferior conditioning in children, adolescents, or young adults could be achieved without TBI (33).

Davies et al. compared HSCT outcomes in children with ALL transplanted from human leukocyte antigen (HLA)-identical siblings who received cyclophosphamide plus TBI conditioning (*n* = 451) vs. those who received oral busulfan plus cyclophosphamide conditioning (*n* = 176) (16). The 3-year probability of overall survival (OS) was 55% [95% confidence interval (CI) 50–60%] with TBI and cyclophosphamide and 40% (95% CI 32–48%) with busulfan and cyclophosphamide (*p* = 0.003), with a higher risk of treatment failure (relapse or death) in the busulfan group [relative risk (RR) 1.39; *p* = 0.017].

A retrospective European Society for Blood and Marrow Transplantation (EBMT) study assessed the role of TBI in patients aged 2–18 years who were transplanted for ALL in remission with a bone marrow or peripheral blood graft from a compatible donor, and compared patients who had received TBI-based myeloablative conditioning (*n* = 1,336) with patients who had been transplanted after chemoconditioning (*n* = 210) between 2000 and 2012 (18). An inferior outcome was reported after chemoconditioning for patients with ALL in second complete remission (CR2), with a 1.75-fold higher risk of death, 1.86-fold higher risk of any failure and a 1.9-fold higher risk of relapse compared with those receiving TBI-conditioning. Conversely, no difference could be detected for those transplanted in first complete remission (CR1). Nevertheless, as TBI was the standard regimen, a selection bias could have affected regimen allocation, with patients who had experienced severe toxicities and infections prior to HSCT being more likely to being allocated chemoconditioning. Furthermore, logistical issues could have limited timely access to fractionated TBI. Similar results were reported when cord blood units were used as the stem cell source, with TBI being associated with a lower risk of relapse than chemoconditioning (34). Within the Centre for International Blood and Marrow Transplant Research (CIBMTR), attempts to decrease the risk of relapse by intensifying the conditioning of 12 Gy TBI and cyclophosphamide—which included increasing the TBI dose to 13.2–14 Gy and/or adding a second chemotherapeutic agent—were not effective (35).

In the recent international, multicentre, randomised FORUM trial in high-risk ALL patients aged 4–21 years at HSCT, 2-year OS was 91% following conditioning with fractionated 12 Gy TBI and etoposide (*n* = 212) compared with 75% following chemoconditioning (a combination of fludarabine, thiotepa, and either treosulfan of busulfan; *n* = 201; *p* < 0.0001); the 2-year cumulative incidence of relapse and treatment-related mortality were 33 vs. 12% (*p* < 0.0001) and 9 vs. 2% (*p* = 0.0269), respectively (19). The median follow-up at interim analysis was relatively short (2.1 years), but the advantage of TBI was striking throughout all subgroups and randomisation was discontinued, as the stopping rule was reached. Whether longer follow-up and associated insights regarding late sequelae will lead to reassessment of the benefit of TBI remains a question.

Efforts to provide equal outcomes with reduced TBI doses, adapted radiotherapy target volumes or the exclusion of TBI are ongoing. For now, however, based on the results of the FORUM study, TBI (12 Gy in six fractions, given twice per day) is the standard of care for ALL patients ≥4 years old who are eligible for HSCT and have no absolute contraindication to radiotherapy.

## The Importance of Minimal Residual Disease

Minimal residual disease (MRD) consists of a small number of leukaemia cells in the bone marrow detectable by flow cytometry, real-time quantitative polymerase chain reaction (RT-qPCR) or next-generation sequencing (NGS) below a level that can be detected morphologically. MRD is recognised as the strongest independent prognostic factor for disease relapse and survival in frontline and relapse ALL treatment, as well as in the transplant setting (36, 37). Most current protocols stratify patients according to response to treatment, including MRD, which, besides guiding treatment decisions, maintains its predictive value (37–39).

The predictive role of pre- and post-HSCT MRD invariably stands throughout ALL patient groups (38), despite the fact that MRD data are mainly used in real time to modulate immunosuppression tapering and/or discontinuation, possibly associated with the use of cell therapy (donor lymphocyte infusion, cytokine-induced killer cells) (40), or targeted therapy (blinatumomab, chimeric antigen receptor T cells) in the attempt to reduce the relapse risk (41–44). The effectiveness of such immunomodulation cannot be assessed.

It has been suggested that the decision regarding the conditioning regimen could be based on MRD, as defined by means of next generation sequencing (NGS) (45). Such an approach is evaluated in the ongoing prospective study which performs a non-TBI based conditioning regimen in patients ≤25 years old diagnosed with B-cell acute ALL who are pre-HSCT NGS MRD negative (NCT03509961) (46).

The role of the MRD level prior to and after HSCT in children and adolescents is discussed in depth in the publication by Merli et al. (47) in this supplement of Frontiers in Paediatrics.

## Immunosuppressive and ANTI-Leukaemic Effects of TBI

The rationale for inclusion of TBI in the conditioning regimen before HSCT for ALL is two-fold: 1) to eradicate leukaemic cells, and 2) to prevent rejection of allogeneic engraftment through ablation of the recipient's immune system. Radiotherapy targets leukaemic cells in the entire body, including in sanctuary sites where chemotherapy delivery is hampered by perfusion, diffusion and blood-barrier effects. Optimising the immunosuppressive effect of fractionated TBI schedules while sparing normal tissue from injury where possible requires consideration of the combination of total dose, dose rate, fraction size, and overall treatment time. Advances in the clinical radiobiology of TBI inferred from data originating from trials or retrospective data sets have been limited, in contrast to what is the case for many solid malignancies and the associated OAR. This is, in part, explained by the large variability in patient and treatment characteristics within and between studies, as well as by the difficulties in obtaining reliable patient-level dosimetry for tissues and OAR from TBI. All of these difficulties are compounded by the fact that many institutional TBI protocols included numerous temporal adjustments to planning and delivery as well as to the dose-time-fractionation regimens used, which further hamper direct comparisons of disease control and toxicity between series. The lack of consistency in practise patterns, dosimetry and reporting of TBI doses among institutions is documented in the recent surveys of practise patterns of paediatric TBI from the European Society for Paediatric Oncology (SIOPE) and Children's Oncology Group (COG) (27, 28). As a result of these obstacles, much of the radiobiological rationale for current TBI regimens is derived from *in vitro* or experimental animal studies, many dating back to the 1970s and 1980s, and only supported qualitatively by clinical data.

### Dose-Fractionation Biology of Leukaemic and Haematopoietic Cells

*In vitro* radiosensitivity estimates have historically been quantified using the D_0_ value: the dose required to reduce the surviving cell fraction to 37% on the log-linear part of the dose vs. cell-survival curve. Normal haematopoietic cells (mainly lymphocytes in most studies) have D_0_ values between 0.5 and 1.4 Gy, indicating overall high radiosensitivity (48–50). D_0_ values for peripheral blood cells *in vivo* tend to be somewhat higher than *in vitro* values. Studies in animals suggest that there is a small subpopulation of haematopoietic stem cells with higher radioresistance than the overall population (51). In a clinical study, Shank et al. studied peripheral blood cell survival kinetics during hyperfractionated TBI (13.2 Gy in 11 fractions of 1.2 Gy, given three times a day) given before cyclophosphamide as HSCT conditioning in 14 children in remission for ALL and found a D_0_ range of 3.7–5.4 Gy for peripheral blood lymphocytes, without a shoulder in the survival curve (see below), and a D_0_ of 10 Gy for granulocytes (52). Absolute nucleated cell concentration in the bone marrow had dropped to 7–44% of base levels only on the last TBI-day, while marrow myeloid elements decreased continuously. Myeloablative TBI has a prolonged effect on bone marrow recovery, with a 30% decreased marrow cellularity even at 1 year post-HSCT (53).

Leukaemic cell populations have an overall high radiosensitivity with median D_0_ values of 0.74 Gy, usually with a minimal or absent shoulder in the survival curve (54). Specific leukaemic cell types, however, show a wide range of *in vitro* radiosensitivities: wider than that of normal haematopoietic cells (55–59). In a study of 74 children with ALL, B-lineage ALL types proved to be more radioresistant than T-lineage ALL types (60). Monzen et al. performed mRNA expression analysis on a model of radioresistant acute promyelocytic leukaemia cells and found that specific changes in intracellular genetic network profiles were associated with radioresistance in their AML cell line (61).

Fractionation sensitivity, i.e., the total dose adjustment required to maintain a given level of biological effect after changing the dose per fraction or the dose rate, is generally quantified using the α/β value of the linear quadratic (LQ) model (62). Higher values of α/β indicate less sensitivity to dose per fraction/dose rate. Historically, this was quantified by the “shoulder” of the *in vitro* dose–survival curve: a large shoulder indicates large fractionation sensitivity, corresponding to a low α/β value in the LQ model.

Early studies of haematopoietic cells generally showed small shoulders of *in vitro* dose–survival curves, suggesting a limited effect of dose-fractionation (63). *In vitro* studies on ALL cells retrieved from 74 children found that, contrary to previous notions, about a third of B- and T-lineage ALL cell clonogens display a shoulder in the survival curve and possess sublethal radiation damage repair capacity, which is most relevant during fractionated radiotherapy (64). Uckun et al. (64) estimated α/β values ranging between 0 and 101 Gy, and two-thirds of progenitor cells from 34 evaluated cases had an α/β value <5 Gy, indicating a substantial effect of dose per fraction.

A large dose per fraction and/or increased dose rate of TBI will counter the recovery of leukaemic cells between fractions (65, 66) but, obviously, this should be balanced against the potential sparing of normal tissue effects from low fraction sizes/low dose rate. Wheldon and Barrett devised a mathematical model for leukaemic cell kill based on 27 fractionated TBI schedules that are iso-effective for interstitial pneumonitis (IP) risk and applied this to a hypothetical patient population with diverse leukaemic intrinsic radiosensitivities (67). They surmised that many of the current TBI schedules have a similar propensity for leukaemia cure in unselected patient populations. Ideally, a patient's individual leukaemic cell radiosensitivity should be known to select their optimal TBI schedule. However, this would only generate a modest improvement in general cure probability and would benefit mainly outliers with relatively low leukaemic radiosensitivity (67). As research into cellular radiobiology predictive assays generally has failed to impact clinical radiotherapy in other indications, it seems unlikely that *in vitro* cellular assays of the individual radiosensitivity of haematopoietic volumes and leukaemic cell types in a patient before beginning TBI-based conditioning prior to HSCT would be a valuable translational addition to future studies regarding ALL HSCT.

As genotyping increasingly becomes a part of the routine clinical work-up of patients with leukaemia, it is conceivable that putative links between genotypes and the effect of TBI will be discovered in the coming years. Genomics, in particular germ-line single nucleotide polymorphisms, have been studied in 10,000s of radiotherapy patients as a potential cause of inter-individual variability in early and late toxicity after radiotherapy (68). Initial reports were encouraging. However, a large UK validation study in patients with prostate or breast cancer with 2-year clinical assessment of late radiation adverse effects showed that the early literature was dominated by false-positive findings (69). More recently, there is emerging evidence from large studies that sequence alterations may affect adverse events after radiotherapy. Somatic sequence alterations in leukaemic cells could also, in theory, affect the therapeutic effect of TBI. So far, except for a few rare genetic disorders, there are currently no generally accepted and validated genotypes that affect radiotherapy prescriptions in other radiotherapy indications (70).

## Clinical Data on TBI Dose-Fractionation Response

In the 1950s, the discovery that stem cell transplantation could counteract acute mortality from the depletion of blood-forming tissues after TBI injury triggered many studies into the application of HSCT against haematologic malignancies and immunodeficiency diseases in particular (71–73). Experiments in mice showed that extremely high lethal TBI doses of 20–50 Gy or higher were needed to sterilise advanced leukaemia in the body (74), and that the graft-versus-leukaemia effect of infused stem cells was therefore essential for cure when lower TBI doses were applied. The first clinical allogeneic HSCTs were performed with TBI-only conditioning and were largely a disappointment because of disease recurrence, non-engraftment, graft-versus-host-disease- (GvHD) and treatment-related death (75). When up to 10 Gy single-fraction TBI was combined with cyclophosphamide, and immunosuppressive and peri-transplantation care evolved, more patients with acute leukaemia survived (20, 24, 76).

However, the acute and late effects of single-fraction TBI, especially for developing children, became an issue of worry. Peters et al. argued that the therapeutic ratio of the radiosensitive normal tissues vs. the immunosuppressive and anti-leukaemic effects of TBI could be improved by decreasing the single-fraction dose rate (which meant an irradiation lasting up to >10 h for patients) or by dose fractionation (77). The latter was confirmed in a randomised trial (78).

Many different fractionation schedules began to be used (79) and it was difficult to evaluate differences in efficacy because of the multifactorial influence of treatment effects, GvHD and toxicities in cohorts of patients with various diseases and age groups (67). Fractionated doses <9–10 Gy would result in non-engraftment and disease relapse (80, 81). In many instances, lung toxicity was found to be the dose-limiting factor at 2-Gy fractionated 16 Gy TBI (82); it was also diagnosed more frequently after single-fraction TBI than after fractionated TBI in leukaemia patients (83–86). For children, other significant TBI effects such as growth inhibition or cataract formation were reduced by TBI fractionation (23, 87). One fractionation schedule that was applied early on was 12 Gy in six fractions given over 3 or 6 days. To optimise the therapeutic ratio, twice-daily fractionation of doses between 1.5 and 2 Gy to doses ≥12 Gy was estimated to be optimal, while more hyperfractionated schedules with three to four fractions daily seem to have worse anti-leukaemic/immunosuppressive effect as well as being impractical in terms of delivery within working hours while giving healthy tissues the aspired 6-hour recovery period between fractions (54, 88–90). Giving 12 Gy TBI in once-daily fractions of 4 Gy increased acute effects such as mucositis (91, 92). A randomised dose-escalation study comparing 12 Gy TBI over 6 days with 15.75 Gy TBI over 7 days displayed a decreased relapse rate after high-dose TBI but increased rate of non-relapse mortality (NRM), ultimately resulting in equal probabilities of survival (93, 94). In a single-centre ALL HSCT cohort, 12 Gy in six fractions over 3 days was deemed the optimal TBI schedule regarding GvHD occurrence and overall prognosis after variations in TBI dose, dose rate and technical setting had been applied during a span of 12 years (95). Another centre—comparing 10 Gy with 12 and 13.2 Gy TBI given over 3 days—concluded that 10 Gy gave the highest 5-year OS benefit (96). One publication compared 16 TBI studies regarding fractionation and dose rate, the combination of which was recalculated into the biologically effective dose (BED) for leukaemic cells and several OAR (e.g., 6 times 2 Gy with a dose rate 0.16 Gy/min gives a BED_leukaemia_ of 14.2 Gy and BED_lens_ of 42.8 Gy) (97). A high BED_leukaemia_ in the fractionated schedules significantly reduced relapse incidence and increased OS. Shielding for lungs, kidneys and lenses was advised to BEDs ≤15, ≤17, and ≤40–45 Gy, respectively.

Hard conclusions regarding TBI fractionation for ALL specifically are difficult to draw from these studies as they cover different patient and disease categories as well as temporal changes in overall and HSCT-specific treatment protocols. The FORUM trial delivered a conditioning of etoposide combined with TBI as a 6 times 2 Gy TBI schedule given over 3 days and lung shielding at 10 Gy (19). For the moment, fractionated TBI schedules giving doses of 12–14 Gy with lung shielding have been adopted as optimal schedules in ALL HSCT by many paediatric radiation oncology centres (27, 28). Nonetheless, continuous reassessment of TBI optimization is needed as pre-HSCT factors improve and new combinations of chemotherapy with lower doses of TBI are researched (98, 99).

## TBI Dose Rate

The biologic radiotherapy effect of TBI on cells and tissues depends on their inherent radiosensitivity, the micro-environment, total dose, fractionation, overall treatment time, dose rate, dose homogeneity, TBI setup, patient and disease characteristics, and other therapies. TBI with an extended source-surface distance (SSD) setup is institution specific, precluding normalisation of TBI dose and dose rate (100). Published works may report dose rate at the prescription point of a patient's midplane, in the lung or in air. Reported values may represent measured or calculated data, and measurement and calculation methods can differ between centres. These differences must be considered when comparing and interpreting published data. In older studies, TBI was often delivered with cobalt teletherapy and source decay exposed the analysed cohorts to varying dose rates through time (81). In modern extended SSD TBI, the dose rate is chiefly determined by the SSD (through the inverse square law) and the linear accelerator dose rate.

In the 1970s, the most commonly used TBI schedule was 8–10 Gy given at a low dose rate over several hours, to balance treatment effect against toxicities (101). Fractionated TBI was recommended to improve the therapeutic ratio. For leukaemic cell kill and allogeneic engraftment success, fractionated TBI with a higher dose rate is preferable to a lower dose rate (77, 89). In preclinical studies, increased dose rates during TBI improved allogeneic engraftment (102–104). In clinical studies, dose rates of ≤0.04 Gy/min showed increased leukaemia relapses in patients given TBI doses of 8.4–12.5 Gy in 3 days (81). Bone marrow displays a marginally increased sensitivity for fractionation with 1.2- and 2-Gy fractions, and little effect of higher dose rates of 0.8 Gy/min when compared with 0.05 Gy/min in single-fraction TBI (105). At dose rates >0.3 Gy/min, no extra effect for haematopoietic cell damage is expected (106).

Multiple studies have explored the effect of TBI dose rate on toxicity. In preclinical studies exploring single-fraction TBI, dose rate changes in a lower dose rate range had a much greater influence on toxicity occurrence in late responding tissues (especially the lung, kidney and liver), than dose rate changes in the higher dose rate range (101, 106). For late non-hematopoietic tissue effects, this resulted in e.g., an iso-effective dose factor of ±2.4 for a dose rate of 0.02 Gy/min, ±1.5 for a dose rate of 0.1 Gy/min, and ±1.0 for a dose rate range of 1 to >10 Gy/min. Experiments in mice indicate that average dose rate may be more relevant for lung tissue toxicity than instantaneous dose rate (107). At midplane dose rates ≤0.15 Gy/min, fractionation of total dose had a greater sparing effect on late-responding tissues than reduction of dose rate (23, 106). High dose rates of 0.75 Gy/min induced more gastrointestinal damage in dogs after TBI than dose rates down to 0.021 Gy/min, but this effect could be compensated for by fractionation (108). In dogs given autologous HSCT, acute TBI tolerance doses measured as 50% mortality at 7 days were comparable between single-fraction and fractionated TBI (2 Gy three times daily) at exposure rates of 0.02–0.1 Gy/min, but fractionation benefit occurred at a dose rate of 0.2 Gy/min, with tolerance doses of 10.56 Gy (95% CI 9.39–11.74) vs. 13.2 Gy (95% CI 11.36–15.05), respectively (109). In mice, low dose rates of 0.05 Gy/min as compared with 0.8 Gy/min, had a highly protective effect on late lethality in single dose TBI, but this effect diminished or disappeared when TBI was given in 1.2- or 2-Gy fractions (105). These studies exemplify that influence of dose rate on toxicity induction diminishes through fractionation, that fractionation increases tolerance of normal tissues, that dose rate changes in the lower dose rate range (e.g., <0.15 Gy/min) influence late toxicity effects more than dose rate changes in the higher dose rate range (e.g., >0.3 Gy/min), and that average dose rate may be more relevant for biological effect correlation than instantaneous dose rate.

In a BED calculation of 16 clinical studies, it was demonstrated that different dose rates at ≤0.15 Gy/min for fractionated schedules do not induce large BED differences for leukaemic cells and OAR, in contrast to single-fraction schedules (97). Most clinical research into dose rate effects has focused on lung toxicity. In 202 acute leukaemia patients, 8 times 1.65 Gy fractionated TBI given at dose rates of >0.15 Gy/min induced significantly more IP and worse OS than dose rates of ≤0.15 Gy/min when lungs were only shielded by the arms in a bilateral beam setup (IP incidence: 29 vs. 10%, respectively, *p* < 0.01; 1-year OS: 60 vs. 76%, respectively, *p* = 0.01) (110). In studies using fractionated conventional TBI, the impact of dose rates up to 0.15 Gy/min becomes negligible for IP development, as long as the registered lung dose does not exceed 8–9 Gy (111–114). At dose rates of 0.15–0.21 Gy/min, IP risk increased with increasing dose rates in studies with lung shielding of 10–12 Gy for TBI schedules of 12 Gy in 6–8 fractions (115, 116). In a meta-analysis including TBI lung dose rates of 0.03–0.41 Gy/min, dose rate was not significantly associated with IP (117). A high dose rate affects late renal damage inasmuch as it can increase BED to levels above tolerance doses, generating the need for kidney shielding (118–120). Cataract development is related to dose rate, with increasing cataract risk at increasing dose rates between 0.02 and 0.56 Gy/min (121, 122). Although not repeated in all publications, clinical studies show that for dose rate ranges of e.g., 0.04–0.4 Gy/min in a conventional SSD TBI setup, increasing the dose rate increases risk of late toxicities in lungs, kidneys and lenses even for fractionated schedules, generating a need for adequate organ shielding.

Momentarily disregarding the numerous influential variables and inconsistent reports regarding the issue of dose rate, dose rates between 0.04 and 0.15 Gy/min seem to be the most frequently reported option for extended-SSD, fractionated conventional TBI schedules in paediatric ALL patients, albeit with appropriate OAR shielding. Regarding immunosuppressive and anti-leukaemic cell effect, the higher end of this spectrum may be preferable. For more staunch multicentre conclusions, we need comparable schedules, uniform specifications, and complete reporting of all relevant parameters including applied dose rates.

Patient comfort is a factor as well. Delivery of 2-Gy fractions at 0.04 Gy/min requires 50 min beam-on time and motionless patient positioning, which would mean more indications for multiple sedations of >1 h in children.

Improved dose homogeneity and specific OAR dose reduction can be achieved with highly conformal TBI techniques. This, along with fractionation, may allow for more favourable toxicity profiles even with a high instantaneous dose rate. Low dose rates are preserved with an image-guided intensity-modulated radiotherapy (IMRT) technique at extended SSD, deriving midplane dose rates of 0.14–0.19 Gy/min (123). First experiences with this technique show encouraging results for outcome and lung/kidney toxicity, with a 15% dose reduction at these organs (124). With highly conformal source-to-axis distance techniques such as TomoTherapy (a device combining a helical computed tomography (CT) scanner and a linear accelerator) and volumetric-modulated arc therapy (VMAT—rotational IMRT delivered on a standard linear accelerator), instantaneous dose rates are inherently higher (e.g., 0.2 to >10 Gy/min) and are variable during treatment (125–127). The first experiences with TomoTherapy and VMAT TBI (with overall instantaneous dose rates of ±13 Gy/min and ±0.31 Gy/min and instantaneous dose rates around the lung of ±8.4 and ±0.11 Gy/min, respectively), showed promising results in 197 children with regard to outcome and toxicity profiles (128). Centres can opt for a decrease of monitor unit output at the level of e.g., the lungs or pelvis to achieve average dose rates of even <0.06 Gy/min if desired (125, 126). Fractionated TMLI, with greater sparing of dose-limiting OAR, may provide a means to preserve immunogenic and anti-leukaemic effects while conveying highly acceptable toxicity profiles with high instantaneous dose rate (32, 129).

## TBI and Hypoxia

Hypoxia as a cause of radioresistance is a well-known problem in rapidly proliferating solid tumours which outgrow their blood supply. It has not gained much attention in leukaemia research. However, it turns out that the microenvironment in the deeper peri-sinusoidal bone marrow regions (where most of the long-term haematopoietic stem cells reside) is hypoxic, with O_2_ levels <10 mmHg (130–132). Moreover, leukaemic cells have been shown to be markedly hypoxic; hypoxia inducible factor-1α (HIF-1α), a molecular marker of hypoxia, was shown to be overexpressed in leukaemic cells in the bone marrow in paediatric patients with ALL (132, 133). Hypoxia induces chemoresistance and may play a role in the maintenance of MRD (134). The level of hypoxia in some leukaemic cells in the bone marrow is sufficient to cause hypoxic radioresistance. However, there are no data to support that this is a significant clinical problem, and, so far, no interventions directed at modifying the hypoxia of leukaemic cells have been proposed.

## TBI and Radiotherapy Boost of Sanctuary Sites

The central nervous system (CNS) and the testes are protected by barriers that are difficult to penetrate by systemic treatment and have been shown to act as sanctuaries for leukaemic cells with a high risk of local recurrence. Including TBI in the conditioning regimen has the distinct advantage of reaching these sites with the planned treatment.

Radiotherapy can also deliver a higher dose to precisely defined volumes: a so-called boost. Adding a radiation boost to the sanctuary sites in order to reduce the recurrence risk was performed often in the past. However, the effectiveness of the systemic regimens has improved very significantly, making radiation boosts unnecessary in most cases (135–141).

The risk of CNS relapse after HSCT is very high in patients with residual CNS leukaemia after chemotherapy or in patients who develop a relapse involving the CNS. For these patients, additional CNS-directed radiotherapy is often considered (140, 142). Most often, whole brain radiotherapy has been applied to a cumulative cranial dose of 18–24 Gy (140). However, data indicate that craniospinal irradiation (CSI) may be more effective, which seems logical with leukaemic cells circulating in the cerebrospinal fluid. CSI is given to a cumulative dose of 18 in 2 Gy fractions (143). CNS-directed radiotherapy is given in the days immediately prior to TBI.

With modern systemic therapy for ALL, testicular relapses are rare. A boost is only considered for patients with a very high risk of testicular relapse, typically patients with residual disease after chemotherapy or who develop testicular recurrence. The scrotal content including both testes (or the contralateral testis after orchiectomy) is irradiated. If only the contralateral testicle with no evidence of disease is present, a single dose of 4 Gy is often given; however, if one or both testes are clinically involved, the cumulative dose (together with TBI) is 18–24 Gy given in 2 Gy fractions in the days immediately prior to TBI.

## TBI Toxicity

Survivors after HSCT can develop morbidities in any organ system and have higher morbidity and mortality rates than those observed in the general population or in non-transplanted childhood cancer survivors (5, 6, 144–146). Very young children (aged below 3–4 years) are more prone to developing serious side effects from HSCT and TBI-based conditioning (4, 147, 148). Concerns are i.e., more negative effects on neurocognition, growth, endocrine and metabolic functioning and second malignancies, and many centres now avoid TBI in these young patients (27). Radiotherapy can cause toxicities depending on patient-, tissue-, disease-, treatment-, dose-, and location-related factors (149). Although many factors are at play in the establishment of HSCT sequelae, TBI-based conditioning causes more late effects than chemoconditioning (4, 5, 150). Within the French Leucémies de l'Enfant et l'Adolescent (LEA) cohort, at a median follow-up of 10.1 years, the 174 patients who received TBI reported more complications than the 66 patients conditioned with busulfan during the same time period (3.01 vs. 2.35, respectively, *p* = 0.03) (151).

Since late effects of HSCT will be explored in another review within the current Frontiers in Paediatrics supplement, this chapter will focus on fractionated TBI effects. Table 1 narrates several fractionated TBI-related sequelae. General observations from the literature are given as well as noteworthy specific articles. Consequences/recommendations for TBI, or consequences after TBI are remarked upon.

**Table 1:** Fractionated TBI related effects after HSCT.

**Acute toxicities**	**References**
During and in the days to weeks after myeloablative TBI, patients can suffer from toxicities such as parotitis, nausea, vomiting, diarrhoea, xerostomia, mucositis, oesophagitis, skin erythema, headache, alopecia, loss of appetite, and fatigue.	(152, 153)
**Consequence for fractionated TBI**
These effects are generally transient. Supporting measures during hospitalisation such as dexamethasone, supplemental IV fluids and antiemetics, pain medication, and skincare can alleviate complaints.	
**Lung toxicity**	**References**
**Interstitial pneumonitis** (also: idiopathic interstitial pneumonitis/pneumonia syndrome): A potentially fatal semi-acute complication that can develop in days to months after HSCT conditioning, with a peak incidence at 60–90 days post-HSCT.	
**General observations**	
Distinction between idiopathic vs. non-idiopathic pulmonary toxicity in publications is oftentimes ambiguous; standardisation in diagnostic workup and definitions is needed to clearly correlate IP incidence with TBI parameters in children.	(154)
IP occurs more commonly after allogeneic HSCT than autologous HSCT.	(155)
After single-fraction TBI, IP occurred more frequently (occurring in up to 60% of patients) and was associated with 50% fatality in studies in the 1970s.	(156)
Most series assessing IP after fractionated TBI included adult and paediatric patients with different hematolymphoid diseases and HSCT conditioning protocols.	(83, 116, 154, 157–161)
IP incidences in children vary from 0 to 35%, typically with a fatal outcome observed in fewer than 20%.	(14, 111, 115, 155, 162–169)
IP incidence is affected by lung radiation dose. Factors that reduce the BED (such as lower total dose, more fractionation, lung shielding, and lower dose rates) decrease IP risk.	(97, 115–117, 158, 160, 161, 170)
**Specific articles**	
Esiashvili et al. analysed 127 children with ALL who received allogeneic HSCT after TBI-based conditioning in different centres, along with cyclophosphamide, thiotepa, or etoposide. TBI doses of 12 or 13.2 Gy were given as six or eight twice-daily fractions, and lung doses were variable according to TBI set up and mode of shielding. Although study-reported grade 4 and 5 adverse events were not clearly related to reported lung doses, OS was significantly better after mean lung doses of <8 vs. ≥8 Gy (HR 1.85; *p* = 0.043). Lung shielding did not cause higher disease relapse.	(111)
Sampath et al. performed a retrospective review of 1,090 patients in 20 studies assessing 26 TBI-based and chemo conditioning regimens; their IP risk model identified lung dose, total dose, fraction dose, cyclophosphamide dose, and busulfan use as predictive factors for IP. Once-daily fractionated 12 Gy TBI induced an IPS incidence of 11% as compared to 2.3 with 50% lung shielding (*p* < 0.05). No dose-rate effect was observed.	(117)
A 2011 meta-analysis of randomised trials comparing chemoconditioning with TBI-based conditioning (mostly fractionated TBI 11–13.5 Gy with variable amounts of lung shielding of 6–13.2 Gy) for allogeneic HSCT in leukaemia patients found no significant differences for occurrence of IP between these conditioning regimens (RR 1.22, 95% CI 0.79–1.88; *p* = 0.37).	(17)
**Long-term lung toxicities (restrictive/obstructive fibrosis and lung function reduction)**	
Busulfan may be associated with more chronic lung toxicity than fractionated TBI, with restrictive pulmonary disease occurring in up to 75% of busulfan-treated patients after a median of 3 years.	(171–173)
Development of restrictive/obstructive lung disease after HSCT is multifactorial, including the transplant regimen, diagnosis, donor major histocompatibility complex mismatch, chronic GvHD, and time after transplant.	
**Consequence for fractionated TBI**	
Paediatric oncology radiotherapy centres reduce the dose given to the lungs, mostly to a mean dose of 8–10 Gy.	(27, 28)
**Liver toxicity**	**References**
Sinusoidal obstructive syndrome (SOS) is a semi-acute complication of allogeneic HSCT with a mean incidence of 14% after HSCT and high mortality rate for severe SOS.	(174–177)
**General observations**	
Numerous HSCT-conditioning chemotherapies, among which busulfan, as well as TBI are associated with SOS.	(175, 176, 178)
Higher SOS incidences may be seen with the addition of other drugs such as sirolimus.	(179)
In preclinical studies and clinical studies in patients with a haematologic malignancy, busulfan and cyclophosphamide conditioning showed more frequent SOS occurrence than TBI conditioning, although both regimens can cause damage to liver sinusoid endothelial cells resulting in SOS.	(17, 33, 180, 181)
**Specific articles**	
In a retrospective analysis of 305 leukaemia patients, as well as in a trial of 157 hematolymphoid malignancy patients with randomised TBI fractionation and dose rates, investigators found no relationship between use of single-fraction 10 Gy vs. fractionated 12 Gy in six fractions or different dose rates and SOS incidence.	(113, 182)
Girinsky et al. found a significantly higher 8-year incidence of SOS after single-fraction 10 Gy TBI (*n* = 73; 14%) vs. fractionated 14.85 Gy TBI (*n* = 74; 4%; *p* = 0.04) in a randomised trial of TBI in adult patients with haematologic malignancies.	(112)
**Consequence for fractionated TBI**	
In dose-escalation studies of fractionated TBI, SOS was the dose-limiting toxicity at 16 Gy in 2-Gy fractions twice per day, or 14–14.4 Gy in 1.2- to 1.6-Gy fractions three times per day.	(82, 163, 170)
A dose reduction of 10% of 14 Gy over the liver was associated with a lower risk of fatal SOS after fractionated TBI in one study (3/20 patients without shielding had fatal SOS vs. 5/98 patients with shielding) without an apparent reduction in engraftment (96%).	(170)
It is unclear whether shielding the liver during TBI increases leukaemia relapse risk.	
**Renal toxicity**	**References**
Chronic renal disease (CRD) occurs in ~17% of patients after HSCT (reported range 3.6–89%) and has multiple risk factors including acute renal failure, GvHD, type of transplant, sex, age, TBI (single-fraction vs. fractionated), impaired baseline renal function, long-term cyclosporine, nephrotoxic drugs, and development of SOS.	(183–185)
**General observations**	
Children are less likely to experience CRD after HSCT than are adults. In a cohort of 148 patients surviving 2 years after HSCT, 12% of 91 adults had CRD vs. 0% of 57 children aged <15 years old.	(186)
Fractionated TBI is variably reported as risk factor in children.	(118, 166, 187–189)
Radiotherapy-related CRD develops in different stages and is caused by pathological mechanisms such as inflammation, fibrosis, and vasculopathy.	(190)
**Specific articles**	
Ellis et al. calculated a pooled odds ratio for CRD of 2.56 for TBI doses >11 Gy from seven combined cohorts in a meta-analysis.	(183)
Based on a meta-analysis, Kal et al. advised to keep the BED <16 Gy, by shielding of the kidneys if needed, to keep the risk of TBI-related CRD below 3%.	(97)
Igaki et al. treated 109 adult and paediatric leukaemia patients with 12 Gy TBI in six fractions with and without kidney shielding; while 2 year survival rates were not significantly different between arms, patients without shielding experienced 21.5% renal dysfunction at 2 years compared with 0% of patients after shielding.	(191)
Lawton et al. performed 14 Gy fractionated TBI on 157 adult patients with various hematolymphoid diseases, with varying amounts of shielding and found lower rates of post-HSCT CRD when higher amounts of shielding were used (actuarial risks of CRD at 2.5 years were 29 ± 7% SE with no shielding, 14 ± 5% with 15% shielding, and 0 with 30% shielding).	(192)
**Consequence for fractionated TBI**	
Dose reduction to the kidneys to a BED <16 Gy should be considered to reduce the risk of CRD.	
**Cataracts**	**References**
Lenses are very radiosensitive and cataracts frequently develop after TBI-containing conditioning for HSCT.	
**General observations**	
Cataract development is more common after single-fraction TBI than after fractionated TBI and is related to dose rate.	(121, 122)
TBI when given as 12–14.4 Gy in six to eight fractions is associated with fewer occurrences of cataracts than when given as 12 Gy in four fractions.	(160, 193)
**Specific articles**	
In 2,149 patients in the EBMT registry, Belkacemi et al. reported a 10-year estimated cataract incidence of 60% after single-fraction TBI (6–11.8 Gy), 43% after fewer than six fractions, 7% after more than six fractions (8.5–16 Gy) (*p* < 0.001), 30% with dose rate ≤0.04 Gy/min, and 59% with dose rate >0.04 Gy/min (*p* < 0.001).	(121)
In a study of 174 paediatric patients with acute leukaemia who received HSCT, cataract incidence after a median of 10 years' follow-up was 51.7%, and most patients received 12 Gy TBI in six fractions.	(151)
A meta-regression model included 1,386 patients from 21 series in which TBI was administered to a total dose of 0 to 15.75 Gy in single-fraction or fractionated schedules and dose rates of 0.04–0.16 Gy/min. Dose, dose × dose per fraction, paediatric status instead of adult, and standard follow-up by an ophthalmologist were predictive of 5-year cataract incidence after HSCT.	(194)
In a model established from 17 reports, Kal et al. calculated that the risk of development of a severe cataract needing surgery was <5% if lens BED was <40 Gy.	(195)
Few paediatric radiotherapy centres apply eye shielding during TBI, although partial shielding did not increase risk of CNS recurrence in a study of 188 children receiving single-fraction 5–8 Gy or two fractions of 6 Gy TBI.	(27, 196)
**Consequence for fractionated TBI**	
Dose reduction to the lenses to a BED <40 Gy should be considered.	
**Endocrinopathies**	**References**
**General observations**	
Endocrine dysfunctions have a high prevalence after allogeneic HSCT, even without TBI.	(197)
The most commonly reported endocrine deficiencies after HSCT are growth hormone deficiencies, subclinical or overt hypothyroidism, metabolic dysregulation, and pre- or post-pubertal gonadal failure. TBI may cause disturbances throughout hormonal axes, from the pituitary to secreting organs.	
Various researchers did not find significant differences in the rate of endocrinopathies between those paediatric patients receiving fractionated TBI vs. chemoconditioning before HSCT, e.g., In a retrospective multicentre study of paediatric recipients of HSCT with a median follow-up of 10.1 years, Bernard et al. found higher incidences of hypothyroidism for TBI-conditioned patients than busulfan-conditioned patients (28.2 vs. 15.2%, respectively, *p* = 0.04), and equivalent gonadal dysfunction (53.9 vs. 48.1%, respectively, *p* = 0.47), but any significant influence of TBI disappeared in multivariate analysis.	(151, 198–200)
Other studies found more endocrine abnormalities after fractionated TBI than after chemoconditioning, e.g., In a single-centre study after a median follow-up of 13.1 years, significantly more endocrinopathies were observed in 23 children conditioned with TBI than in 17 children receiving chemoconditioning (≥1 endocrine deficiency: 91 vs. 41%, respectively, *p* < 0.05).	(12, 164, 201–205)
Metabolic syndrome, insulin resistance, and abnormal glucose tolerance can occur in HSCT survivors in the absence of obesity; related factors such as increased waist-to-hip ratio, abnormal glucose tolerance, fasting hyperinsulinemia, diabetes mellitus, dyslipidaemia, and hypertriglyceridaemia have been observed in retrospective studies in inconsistent numbers and relationships to TBI.	(206–208)
**Consequences after fractionated TBI**	
With increasing age of childhood ALL survivors receiving allogeneic HSCT, disturbances in endocrine systems and the metabolic syndrome spectrum should be monitored and corrected where possible.	
**Growth impairment**	**References**
**General observations**	
Childhood ALL survivors are at risk of growth impairment, especially when treated before puberty, after receiving higher-dose cranial radiotherapy (≥20 Gy) or radiotherapy to the spine, and girls are more at risk after gonadal failure.	(209)
TBI is associated with growth impairment through growth hormone reduction and a direct effect on bone growth plates; the latter occurs mainly after radiation doses of more than the equivalent of 15 Gy in 2-Gy fractions (EQD2).	(210, 211)
Final height can be diminished by −1.0 to −2.5 standard deviation scores compared to the average height of the population or the expected final height calculated from parental heights.	(212–214)
Younger children are more greatly affected than older children, and single-fraction TBI causes a greater decrease in final height than fractionated TBI.	(87, 212–215)
Even after fractionated TBI, the majority of patients (>75%) reach a final height within the normal range of the average population.	(87)
**Consequences after fractionated TBI**	
Growth hormone treatment has a positive effect on growth rate and final height but does not induce a “catch-up effect” and may be less effective in ALL patients than in children receiving HSCT for other reasons.	(216–218)
**Cardiovascular complications**	**References**
**General observations**	
After HSCT, endothelial damage is induced by conditioning regimens with or without TBI and by HSCT complications such as GvHD.	(219, 220)
Patients receiving HSCT have a higher prevalence of metabolic syndrome and atherosclerosis than general, both of which predispose to cardiovascular adverse events such as myocardial infarction, stroke and peripheral vascular disease.	(220–227)
TBI (as compared to chemoconditioning), TBI dose (≤10 vs. >10 Gy) and TBI fractionation (single-fraction vs. multiple fractions) were not associated with direct cardiovascular outcomes in several studies.	(228, 229)
However, use of TBI conditioning and a higher TBI dose both emerged as risk factors for cardiometabolic traits such as metabolic syndrome, higher fasting insulin, higher blood pressure, adverse lipid profile, subclinical decreased systolic and diastolic heart function, and higher waist-to-hip ratio in studies that followed children after HSCT.	(203, 206, 223, 230–235)
**Specific articles**	
Accumulated data in 24,215 patients on cardiovascular disease risk 5 years after treatment for childhood cancer show an increase in clinically manifested cardiac sequelae decades after radiotherapy: low-to-moderate radiotherapy doses (5–19.9 Gy) to large cardiac volumes (≥50% of the heart)—as is true for TBI—were associated with an increased rate of cardiac disease (relative rate 1.6, 95% CI 1.1–2.3) compared with no cardiac radiotherapy.	(236)
**Consequences after fractionated TBI**	
With prolonged follow-up, TBI-treated patients are at risk for cardiovascular adverse events and should be chronically monitored to ameliorate risk factors where possible.	
**Neurocognitive effects**	**References**
It is difficult to compare studies of neurocognitive function with one other. Different study methodologies, patient characteristics, treatment schedules, use or lacking of baseline testing, comparisons with control groups, and the length and manner of follow-up hamper direct comparisons. Moreover, cognitive function does not always directly relate to educational functioning.	(237, 238)
**General observations**	
Regarding paediatric leukaemia patients who received radiotherapy only in the form of single-fraction or fractionated TBI before HSCT, studies report mostly clinically insignificant but statistically significant decrements in intelligence quotient (IQ) or sensory-motor and cognitive functioning, with however profound effects in children receiving TBI before the age of 3–4 years. This is one of the main reasons to refrain from TBI at such young ages.	(147, 148, 237, 239–243)
In contrast, various studies of patients with mixed diagnoses found no significant changes in children's cognitive status after HSCT, even with TBI.	(244–247)
The difference may be the additive adverse effect of methotrexate therapy. Even in children with ALL treated without radiotherapy, IQ deficits of 6–8 points and deficits in several neurocognitive domains as compared with healthy controls are frequent.	(248, 249)
**Specific articles**	
The PENTEC group recently modelled the detrimental interaction between cranial radiation and methotrexate. Methotrexate increased the risk of an IQ <85 to a level equivalent to a generalised uniform brain dose of 5.9 Gy; this effect should be added to any received cranial radiotherapy dose in the PENTEC risk computation model.	(250)
A recent study by Zajac-Spychala et al. evaluated differences regarding neuropsychological outcomes and anatomical changes on MRI at a median of 5 years after therapy between paediatric patients with high-risk ALL who were treated with or without HSCT with fractionated TBI, and newly diagnosed ALL patients. Transplanted patients had significantly lower volumes of white and grey matter and lower cognitive performance in several neuropsychological domains than the non-transplanted patients. This underlines the added detriment of TBI-based HSCT in high-risk ALL patients.	(251)
**Consequences after fractionated TBI**	
An expert review from the CIBMTR and EBMT on the neurocognitive dysfunction in both adult and paediatric HSCT recipients recommends neurocognitive testing in children before and 1 year after HSCT and then at the beginning of each new stage of education.	(238)
The vast majority of these children will still display neurocognitive functioning skills within the average population range and their very-long-term neurocognitive quality of life is likely to be only moderately affected.	(252)
**Secondary malignancies**	**References**
Second malignant neoplasms (SMNs) are a distressing complication for childhood ALL survivors. Children who have received HSCT form a special risk category.	(253–257)
**General observations**	
Chronic GvHD may have influence on the risk of SMN but this has not been systematically observed.	(204, 258–260)
Prolonged immunosuppression may play a role in the correlation between chronic GvHD and SMN.	(261)
**Specific articles**	
In a cohort of 3,182 childhood acute leukaemia survivors who underwent HSCT, 25 solid tumours and 20 post-transplant lymphoproliferative disorders were observed after a median of 6 years (range 0.4–14.3 years). The cumulative risk of solid cancers increased to 11% at 15 years and multivariate analyses showed increased risks of solid tumour associated with high-dose TBI of ≥10 Gy as a single fraction or ≥13 Gy as a fractionated dose, and younger age (especially <5 years old at transplantation).	(260)
In a study of 426 children after allogeneic HSCT for multiple indications, 18 out of 20 SMNs occurring at a median follow-up of 11.7 years (range 5.4–21.5 years) developed after 12–14.4 Gy fractionated TBI.	(255)
A study of 826 adolescents and young adults who received HSCT for AML extrapolated a 10-year cumulative incidence of SMN of 4%, which was equally distributed between those patients conditioned with TBI or chemotherapy; 16 tumours were diagnosed after a median follow-up of 77 months (range 12–194).	(262)
**Consequences for after fractionated TBI**	
All HSCT recipients and their caregivers should be advised about SMN risks and undergo appropriate screening based on the patient's predisposition.	(261)
**Additional late effects**	**References**
Additional late effects occurring in patients who received TBI conditioning before HSCT in childhood include oral/dental sequelae, potential splenic dysfunction, changes in body mass index, and body composition and musculoskeletal complications.	(263–267)
ALL survivors should be followed for late effects according to appropriate risk-based protocols in long-term screening programs.	(5, 268)

*AML, acute myeloblastic leukaemia; ALL, acute lymphoblastic leukaemia; CIBMTR, Centre for international blood and marrow transplant research; CNS, central nervous system; CRD, chronic renal disease; EBMT, European society for blood and marrow transplantation; GvHD, Graft versus host disease; HSCT, haematopoietic stem cell transplantation; IQ, intelligence quotient; MRI, magnetic resonance imaging; OS, overall survival; PENTEC, Paediatric normal tissues effects in the clinic; SMN, secondary malignant neoplasm; SOS, sinusoidal obstructive syndrome; TBI, total body irradiation*.

## Setup and Planning for Conventional and Highly Conformal TBI Techniques

TBI practise worldwide remains varied, with radiotherapy centres typically developing site-specific setups and techniques (25–28, 79, 269, 270). Conventional TBI is mostly delivered using extended SSD techniques (79), where the radiation beam covers a patient's entire body, and delivers a relatively low dose rate in the patient as a consequence of linear accelerator dose rate adjustability and the inverse square dose reduction with distance (Figure 1). Other setups can be multiple parallel or adjacent beams, sweeping beams, a moving couch underneath a static beam, and field-in-field techniques (271–273).

**Figure 2:**
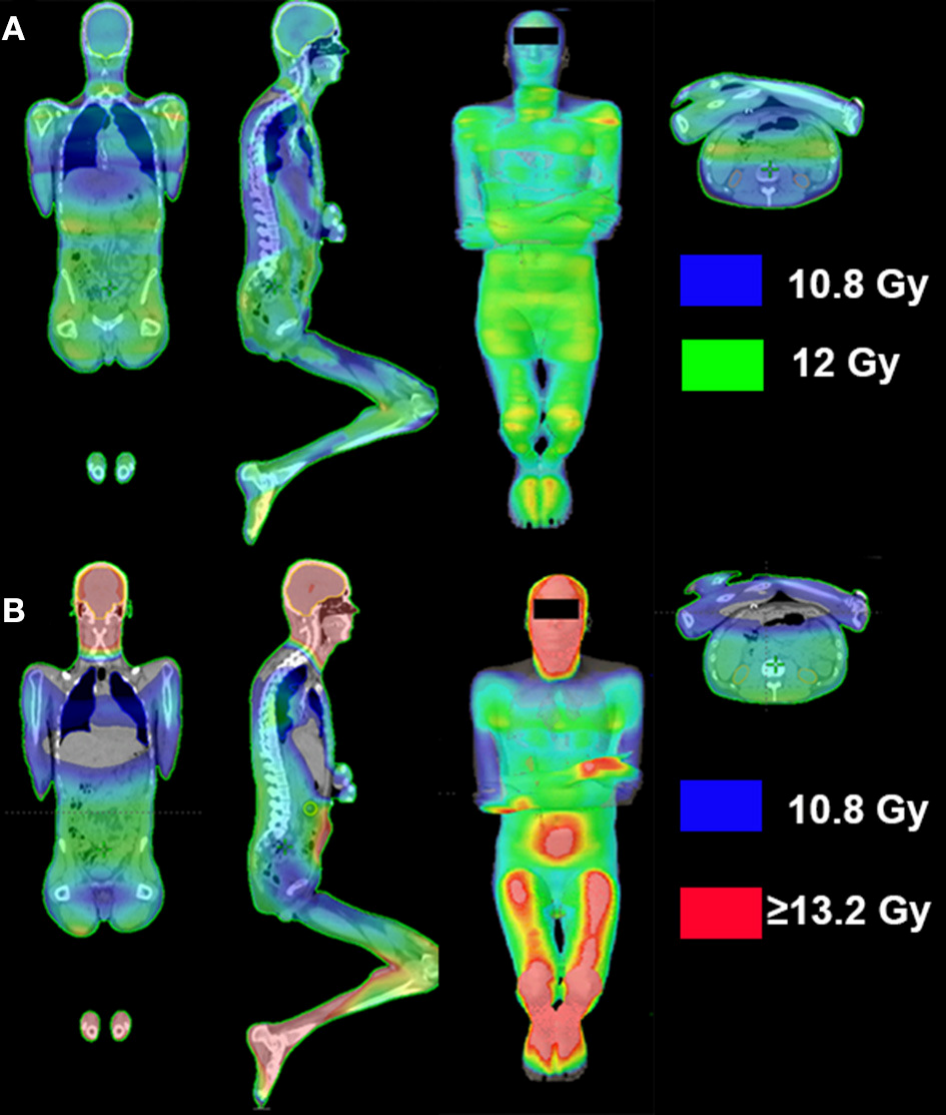
Conventional vs. SSD IMRT-planned total body irradiation dose distribution. **(A)** Computed tomography (CT)-planned, image-guided intensity-modulated radiotherapy (IMRT) dose distribution with lateral-beam setup at source-surface distance (SSD) (123); dose reductions were planned over lungs, and kidneys. **(B)** CT-planned two-dimensional conventional total body irradiation dose distribution with a lateral-beam setup, with lung dose reduction using lung blocks; the isowash-depicted dose range in the images represents 90% (10.8 Gy; blue) to ≥110% (≥13.2 Gy; red) of the prescribed dose.

Many large, open-field conventional techniques result in rather heterogeneous dose distributions, delivering between <80% to even >120% of prescribed doses (Figure 2B), although efforts are made to reduce heterogeneity to within 10%, according to guidelines (e.g., the American Association of Physicists in Medicine guidelines, Report No. 17) (274). The last decade has seen nascent implementation of highly conformal isocentric techniques (where the radiation gantry rotates around the patient on the treatment couch), with the intention to improve dose distribution homogeneity and to reduce the dose to OAR.

### Extended SSD Treatments

#### The Use of a Treatment Planning System

Several clinics deliver TBI plans calculated without the use of a treatment planning system (TPS) (25). These non-TPS based techniques may have remained unchanged for decades and do not allow for the determination of dose-volume histograms of the body or the OAR to be evaluated the way they would be in mainstream radiotherapy practise. Only large open-field TBI treatments should be calculated by these manual workflow methods. It is worth noting that dose estimates made without the use of a TPS may be quite inaccurate: this makes interpretation and comparison of older published outcomes such as dose-response relationships, dose rates, and normal tissue tolerances difficult.

If an isocentrically commissioned TPS is used at extended SSDs, the accuracy of the TPS must be verified for that specific geometry because the beam may have a different energy spectrum, resulting in a change in the depth-dose distributions and a larger component of in-room scatter to the patient dose (275). If verified under extended SSD conditions, an isocentric TPS can be used to calculate dose distributions from more complex techniques such as “step and shoot” IMRT (123, 155, 276) (Figure 2A) or extended SSD VMAT (277, 278).

#### Beam Angles, Spoilers, and Tissue Compensators

In extended SSD TBI, beams are typically delivered using an opposed anterior-posterior (AP-PA) technique, bilateral technique (Figure 2B), or combination of the two (271). Recent data showed that the use of a solely bilateral technique in children is disadvantageous since it results in higher lung doses and decreased survival (111).

Also in extended SSD TBI, a beam spoiler, usually a 1–2 cm thick acrylic screen that is placed in front of the patient, is typically used to counter the skin- and subcutaneous tissue-sparing effect of photon beams (279) (Figure 1). Depending on the protocol, tissue compensators that provide tissue-equivalent dose attenuation may be required to improve dose homogeneity across narrow body sections (155).

#### Lung Shielding

In TBI delivered with large open fields, the lung dose will be greater than the dose to the rest of the body because of the lower density of lung tissue (280). Shielding can be used to reduce the lung dose to the prescribed dose (280–282) or below the prescribed dose (159, 281, 283, 284) and may be achieved using metallic blocks (159, 284) or multi-leaf collimators with an IMRT setup (123). Unavoidably, lung shielding also reduces the dose to the target tissues surrounding the lungs, such as bone marrow in the ribs or mediastinal lymph nodes. The dose to these tissues may be increased by electron boost fields and mediastinal photon fields, respectively (285, 286).

Another issue when using a TPS is that it may not account for the scattered electrons from the non-shielded areas (287), which may increase the actual lung dose considerably. However, TPS algorithms have evolved. The differences between dose distributions calculated by the pencil beam and anisotropic analytical algorithms can be considerable (288), and thus discrepancies between measured doses and doses calculated with a pencil-beam algorithm may not be relevant to modern practise.

#### Shielding of Other Organs

While lung shielding for paediatric TBI delivery is common practise for many clinics (Figures 1C,I) (27, 28), shielding of other organs occurs infrequently. However, shielding should be considered for kidneys and lenses in children (Figure 1C). Dose-effect evaluation of 14 published cohorts produced a kidney BED tolerance threshold of 16 Gy (195). This report and others concluded that kidney shielding is required to avoid post-TBI CRD for almost all myeloablative regimens (192). Eye shielding for cataract reduction has been discussed in several papers (121, 122, 151, 160, 193). Eye shielding to BED <40 Gy reduces the risk of severe cataracts and increases latency time of cataract formation (195, 196).

Individual centres have conventional TBI setup protocols for shielding of the heart, liver (170) and even ovaries (289) but these measures are reported incidentally and no clear recommendation can be given. With highly conformal techniques, centres may choose to deliver reduced doses to multiple OAR, while the bone marrow/lymphoid target volume is adequately covered (31, 124).

### Isocentric Highly Conformal TBI Techniques

**Figure 3:**
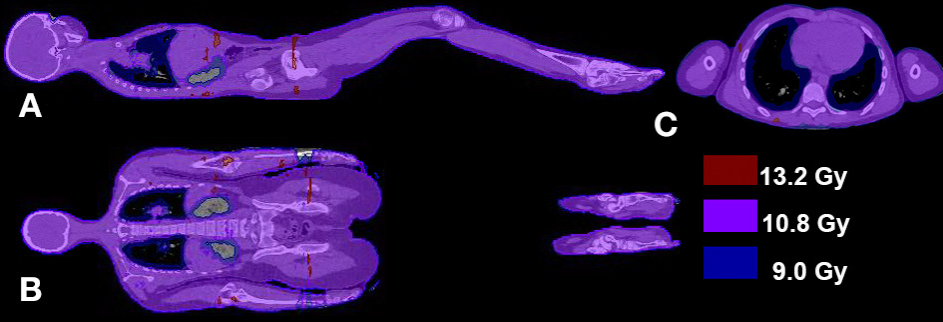
CT-planned VMAT total body irradiation technique dose distribution. Computed tomography (CT)-planned volumetric-modulated arc therapy (VMAT) total body irradiation technique dose distribution for a 12 Gy prescription dose in the sagittal **(A)**, coronal **(B)**, and transversal view **(C)**. The isofill-depicted dose levels are 75% (9 Gy; blue), 90% (10.8 Gy; purple), and 110% (13.2 Gy; red) of the prescription dose.

Isocentrically delivered IMRT TBI requires the use of a TPS. It is a fundamentally different approach to extended SSD TBI because it uses a much higher dose rate and requires field junctioning. Examples of isocentric TBI techniques include TomoTherapy (29, 290–293) and VMAT (30, 125, 127, 294) (Figure 3). These isocentric techniques are seeing nascent clinical implementation in centres around the world, although outcome data from long-term follow-up are yet to be published.

The challenge of field junctioning in these techniques includes the combination of head-first and feet-first treatment, as the couch travel ability of linear accelerators is limited to 120–150 cm (295). Most centres plan five to nine isocentres along the patient's longitudinal axis (30, 125, 126, 294). Aspects that have to be considered include dose homogeneity in the junction areas, junction from head-first to feet-first treatment and robustness of the dose in junction areas.

Modern TPSs allow the combined optimization of multiple isocentres and, thus, homogeneity constraints will automatically include junction areas. This issue has been extensively addressed in the context of CSI, which has even more challenging homogeneity requirements (296–298). Special complexity in TBI results from isocentre extension over two separate datasets with different patient orientations. This is handled either by a mutual “bias dose” addition in each plan orientation (30, 127) or by the use of help contours to create decreasing or increasing doses in the transition areas (294, 295). If inhomogeneities resulting from missing divergence compensation are accepted, legs can also be treated with a simple AP-PA technique (125).

At junction areas, robustness against setup errors is primarily determined by two factors: 1) the length of the field overlap, and 2) the dose profile in the transitional region (299, 300). Whereas the former can be easily addressed by the choice of position and number of isocentres, the latter is largely influenced by the optimization and segmentation algorithm of the TPS and can be supported by techniques such as “gradient optimization” (299). In order to retain the planned inter-isocentre distance, setup corrections must never be made for single isocentres only but always for the entire beam set. This is substantially complicated by the length of the planning target volume (PTV): small rotational errors can produce significant lateral shifts in parts of the body. Thus, planning has to ensure PTV coverage with regard to setup as well as geometric and intrafraction motion uncertainties. Whereas open-field techniques imply an inherent robustness against those errors, robust VMAT planning is more challenging and—once again—is dependent on the TPS. In principle, the complexity of segments should be limited and field borders should be extended from the surface, which can be supported by the use of a virtual bolus (29, 292, 301). The prescription of the skin dose has to be handled carefully as some TPSs tend to compensate dose build-up with small highly weighted tangential fields (302). Usually, the PTV is contracted to 5 mm below the skin (30, 127) but, in practise, the combination of multiple arcs, oblique beam incidence and beam exit from all angles significantly reduces the intrinsic photon beam skin-sparing effect (31).

### Other Physics Aspects

#### Energy

With both isocentric and extended SSD techniques, the choice of energy is pertinent. A beam energy of 6 or 10 MV does not produce an additional neutron dose to the patient or staff. For bilateral TBI setups, photon intensities of at least 10 MV provide more homogeneity than do lower intensities; homogeneity can increase with 18 to 24 MV beams but this is relevant mostly for patients with greater body diameters (162).

#### Treatment Imaging

If shielding or non-open fields are used for TBI delivery, treatment imaging may be used to monitor the position of the patient relative to the fields or the position of the shielding relative to the patient (123, 275).

The accuracy requirements of image guidance depend on the plan complexity. They are generally higher for highly conformal techniques and precision OAR dose reduction. Isocentric techniques require multiple images to cover at least part of the whole-body PTV but optical surface-guided devices might also be used (127). The beam size poses an additional challenge in extended SSD techniques: positioning the imager in the treatment beam requires considerable shielding to protect the electronics from radiation damage. Image acquisition using the megavoltage beam with a detector positioned downstream from the patient may facilitate online verification of organ shielding but the relatively poorer image resolution has to be taken into account.

#### *In vivo* Dosimetry

*In vivo* dosimetry allows the delivered dose to be monitored to ensure that it is sufficiently close to the prescribed dose, making it possible to adjust the fractional dose if needed. Possible measurement devices include diodes, thermo-luminescent dosimeters, optically stimulated luminescence dosimeters, ionisation chambers, and film (303). These devices have varying sensitivities to temperature, orientation with respect to the direction of the radiation, beam energy, and radiation exposure. Some devices offer instantaneous read-out while some do not. Their readings may differ somewhat (304). Dosimeters may be used to measure dose at the patient surface (at the beam entry and/or exit). The dose at that level within the patient must then be extrapolated from these measurements.

While the uncertainty in the measured dose in TBI may be considerable, *in vivo* dosimetry facilitates a check on the delivered dose. This is particularly pertinent when introducing a new technique or when not using a TPS.

## Organ Sparing Total Body Irradiation, Total Marrow Irradiation, and Total Lymph Node Irradiation

Image guided highly conformal delivery of TBI allows the radiation oncology and the transplant teams to define what critical organs to spare, what anatomic structures to target, and the dose that each organ and target structure should receive. This offers the advantage to reduce acute and long-term toxicities (305), the potential to reduce risk of secondary malignancies (306), and the ability to dose escalate to target structures with acceptable toxicities and improved outcomes (307). This is particularly relevant to the paediatric population where, in patients with ALL receiving fractionated TBI, mean lung dose ≥8 Gy was associated with a statistically significant decrease in overall survival (111).

**Figure 4:**
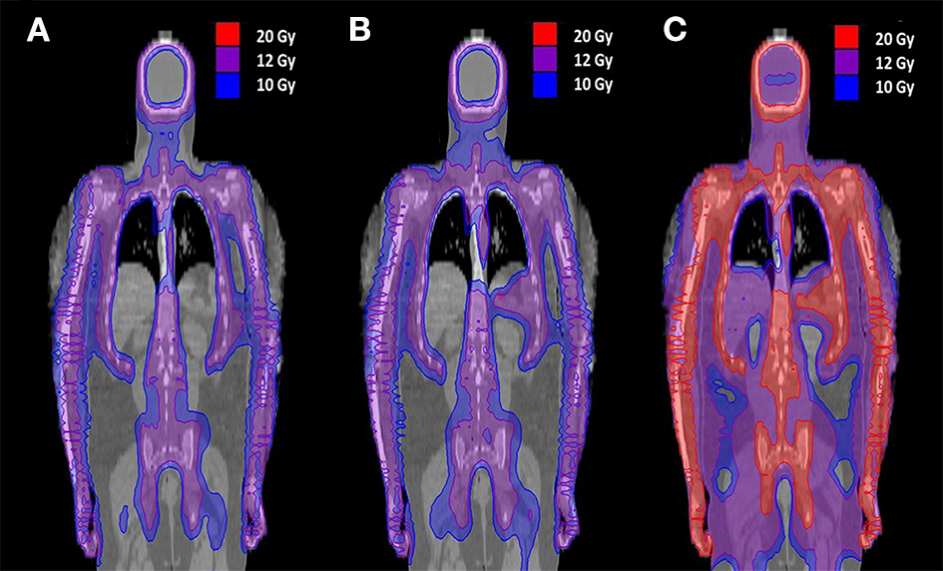
Radiation dose distribution in the coronal plane of TMI and TMLI with different TMI/TMLI approaches. **(A)** Total marrow irradiation (TMI) of 12 Gy to the bone marrow. **(B)** Total marrow and lymphoid irradiation (TMLI) of 12 Gy to bone marrow and the lymph nodes. **(C)** TMLI of 20 Gy to the bone, spleen, and lymph node chains, with a liver and brain prescription dose to 12 Gy. The isofill-depicted dose levels are 10 Gy (blue), 12 Gy (purple), and 20 Gy (red).

TMI (Figure 4A) and TMLI (Figure 4B) are defined as highly conformal organ sparing forms of TBI delivered to the bone marrow, lymph nodes, and spleen (308–310), while sparing lungs, kidneys, heart, oral cavity, GI tract, and other critical organs. In some studies the liver, brain and testes are included as target regions (Figure 4C) (311). Today the terms TMI/TMLI can be broadly applied to a spectrum of highly conformal IMRT TBI dose distributions, including TBI with only lung sparing, which has been shown to result superior dose reduction to the lungs compared to conventional TBI delivery using lung blocks (312).

**Figure 5:**
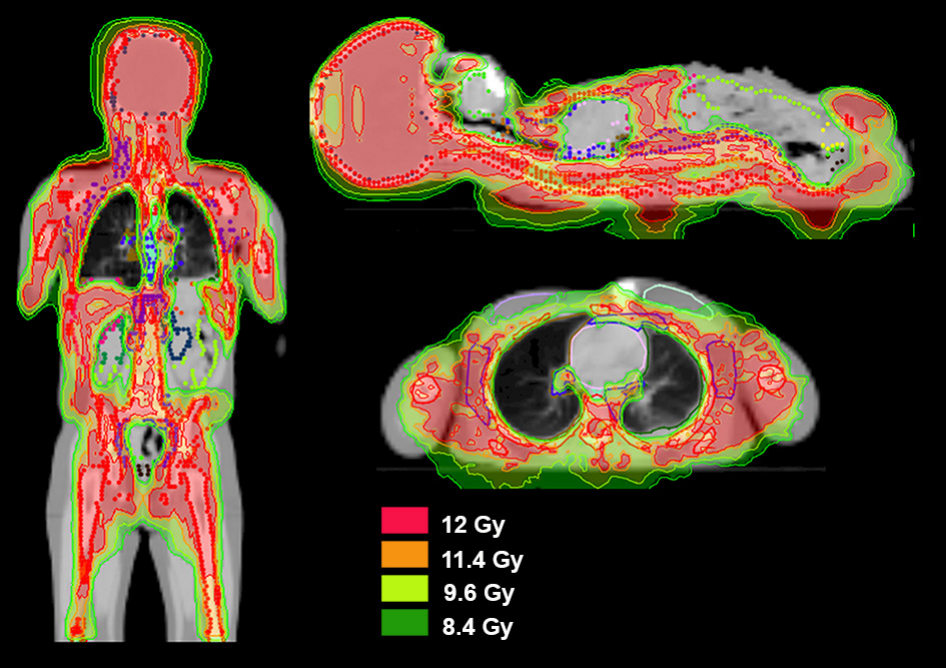
Radiation dose distribution of TMLI in a young patient. Isofill-depicted dose levels of a 12 Gy total marrow and lymphoid irradiation (TMLI) plan in a 5 year old patient with ALL. Target structures were bone, lymph nodes, spleen and brain. The isofill-depicted dose levels are 8.4 Gy (dark green), 9.6 Gy (light green), 11.4 Gy (orange), and 12 Gy (red).

The advantages of IMRT based delivery of TBI and TMI/TMLI are clinically important for both adult and paediatric patients, particularly in patients with co-morbidities who cannot tolerate standard myeloablative TBI regimens, in paediatric patients to limit short and long term toxicities, and in patients with relapsed or refractory (R/R) disease who have no standard transplant options. Figure 5 and Table 2 provide an example of a TMLI plan of a 5 year old patient with ALL, with superior organ dose reduction compared to conventional SSD TBI.

**Table 2:** Median doses (Gy) to organs at risk for conventional TBI with lung blocks vs. TMLI in a 5 year old patient with ALL.

**Organ**	**TBI 12 Gy with lung blocks**	**TMLI 12 Gy (bone, lymph nodes, spleen, liver, and brain)**
Lungs	8.2	4.7
Kidneys	12.0	6.1
Heart	11.1	4.6
Oral Cavity	11.9	2.9
Oesophagus	12.4	3.8
Gasto-Intestinal tract	12.1	3.7
Bladder	12.0	6.4
Thyroid	12.2	3.9
Eyes	11.2	6.2

TMI and TMLI are feasible because of advances in IMRT that have made targeted irradiation of large body regions possible (308, 310, 313–316). The first attempts to cover the whole bone marrow with a very conformal dose distribution were performed with helical TomoTherapy (HT) (308). The first planning studies of HT-based TMI showed that the technique was feasible and that good target coverage could be achieved while reducing doses to key normal tissues by 35–70% compared with conventional TBI (308, 310).

This was followed by the use of a standard linear accelerator to deliver TMI with a number of static (so-called “step and shoot”) IMRT fields (313, 314), with a dose reduction of 29–65% to various OARs in comparison with conventional TBI (314). VMAT-based TMI was shown to obtain comparable target coverage to that obtained with HT and IMRT, with similar dose reduction to normal tissues (314–316).

### Clinical Trials in Acute Leukaemia Including TMI and TMLI

The majority of trials have focused on patients with refractory or relapsed (R/R) AML and ALL and are summarised below and in Table 3. Most clinical trials have included adult and adolescent patients, but the strategies being evaluated are applicable to younger paediatric patients (128). A prospective observational study including 37 children and adults treated with myeloablative TMI of 12 Gy in six fractions over 3 days, found favourable outcomes regarding GvHD- and relapse-free survival, as well as toxicity outcomes when compared with retrospective data of 33 patients receiving TBI (326).

**Table 3:** TMI and TMLI Trials in Patients with Acute Leukaemia.

Study*	**Patients (*N*) age range (years)**	**Disease**	**Radiation targets**	**TMI dose (fractionation)**	**Chemotherapy**	**Outcomes**
Wong et al. (317), Phase I, NCT00540995	20 23–52	Relapsed or refractory AML	Bone, nodes, testes, spleen 12 Gy: liver, brain	12 or 13.5 Gy (1.5 BID)	Bu 4,800 μM*min VP16 30 mg/kg	NRM: 8 of 20 patients CR: 5 of 20 patients at 20.8–49.4 months
Stein et al. (311), Phase I, NCT02446964	51 16–57	AML, relapsed or refractory ALL	Bone, nodes, testes, spleen 12 Gy: liver, brain	12–20 Gy (1.5–2.0 BID)	Cy 100 mg/kg VP16 60 mg/kg	NRM: 3.9% at day 100, 8.1% at 1 year PFS: 40% at 1 year OS: 55.5% at 1 year, 41.5% at 2 years
Stein et al. (311, 318), Phase II, NCT02094794	57 16–59	AML or ALL, IF, relapsed or >CR2	Bone, spleen, node 12 Gy: liver, brain	20 Gy (2.0 BID)	Cy 100 mg/kg VP16 60 mg/kg	NRM: 4% at day 100, 6% at 1 year PFS: 48% at 1 year OS: 67% at 1 year
Patel et al. (319), Phase I, NCT00988013	14 20–65	Refractory or relapsed AML, ALL, MDS, MM, CML	Bone	3–12 Gy (1.5 BID)	Flu 40 mg/m^2^/day × 4 Bu 4,800 μM*min	NRM: 29% RFS: 43% OS: 50%
Hui et al. (320), Phase I, NCT00686556	12 2–55	High-risk ALL, AML CR2, CR3, relapse, IF	Bone	15 or 18 Gy (3.0 BID)	Flu 25 mg/m^2^/day × 3 Cy 60 mg/m^2^/day × 2	NRM: 42% at 1 year Relapse rate: 36% DFS: 22% at 1 year OS: 42% at 1 year
Rosenthal et al. (309), Jensen et al. (321), Pilot, NCT00544466	61 9–70	AML, ALL >50 years old or comorbidities	Bone, nodes, spleen ALL: testes, brain	12 Gy (1.5 BID)	Flu 25 mg/m^2^/days × 4 Mel 140 mg/m^2^	NRM: 30% at 2 years, 33% at 5 years EFS: 49% at 2 years, 41% at 5 years OS: 50% at 2 years, 42% at 5 years
Welliver et al. (322), Pilot, NCT02122081	15 18–75	High-risk AML, ALL, MDS >50 years old or comorbidities and unable to undergo TBI-based regimens	Bone, brain, testes	12 Gy (2.0 BID)	Cy	NRM: 4 of 16 patients Median OS: 313 days
Al Malki et al. (323), Arslan and Al Malki (324), Phase I, NCT02446964	29 21–58	AML, ALL, MDS CR1 high risk, CR2, CR3, refractory Haplo-identical	Bone, spleen, nodes 12 Gy: liver, spleen 16 Gy: testes in ALL 12 Gy: brain in ALL	12–20 Gy (1.5–2.0 BID)	Flu 25 mg/m^2^/day × 5 Cy 14.5 mg/kg/day × 2 PTCy 50 mg/kg/day × 2	NRM: 9.3% at 1 year OS: 83% at 1 year Relapse rate: 24% at 1 year
Pierini et al. (325), Phase II, NCT03977103	50 38–65	AML CR1, CR2, PR Haplo-identical	Bone, nodes	TMLI: bone 13.5 Gy; nodes 11.7 Gy if >50 years old TBI: 13.5 Gy in nine fractions or an 8 Gy single fraction if ≤50 years old	Thio 5–10 mg/kg Flu 150–200 mg/m^2^ Cy 30 mg/kg/day T-cell manipulated graft	NRM: 10 patients Relapse: 2 patients Moderate/severe cGvHD: 1 patient Moderate/severe cGvHD/RFS: 75%
Stein et al. (307), Pilot, NCT03467386	18 19–56	AML CR1 and CR2 Matched donor	Bone, spleen, node 12 Gy: liver, brain	20 Gy (2.0 BID)	PTCy 50 mg/kg/day × 2	Mild cGvHD: 5 patients OS: 100% at 1 year RFS: 80.8% at 1 year NRM: 0% at both day 100 and 1 year Relapse: 3 patients (16.7%)

*NCT numbers are Clinicaltrials.gov identifiers. AML, acute myeloblastic leukaemia; ALL, acute lymphoblastic leukaemia; BID, twice per day; Bu, busulfan; cGvHD, chronic graft-versus-host disease; CML, chronic myeloid leukaemia; CR1, first complete remission; CR2, second complete remission; CR3, third complete remission; Cy, cyclophosphamide; DFS, disease free survival; EFS, event-free survival; Flu, fludarabine; Gy, Gray; IF, induction failure; MDS, myelodysplastic syndrome; Mel, melphalan; MM, multiple myeloma; NRM, non-relapse mortality; OS, overall survival; PFS, progression-free survival; PR, partial response; PTCy, post-transplant cyclophosphamide; RFS, relapse-free survival; TBI, total body irradiation; Thio, thiotepa; TMI, total marrow irradiation; TMLI, total marrow and lymphoid irradiation; VP-16, etoposide*.

#### Dose-Escalated TMI and TMLI

Dose escalation by conventional delivery of TBI has reduced relapse rates but has failed to increase OS because it increases toxicities and non-relapse mortality (NRM) (94, 327, 328), underscoring the need to develop targeted and organ-sparing forms of radiotherapy such as TMI. In a Phase I trial of 51 patients <60 years old with R/R AML and ALL, patients were conditioned with TMLI (12–20 Gy in 10 fractions on days −10 to −6), cyclophosphamide (100 mg/kg on day −3) and etoposide (60 mg/kg on day −5) prior to allogeneic HSCT (Figure 4C) (311). Dose escalation with acceptable toxicity to 20 Gy was achievable (327). NRM rates were 3.9% at day 100 and 8.1% at 1 year. A subsequent Phase II trial in 57 patients reported 1-year estimates of NRM, OS and PFS of 6, 67, and 48%, respectively, which are superior outcomes to those reported for standard-of-care regimens (318).

A Phase I trial of TMI (3–12 Gy delivered as two fractions of 1.5 Gy per day during 1–4 days) with fludarabine (40 mg/m^2^/day × 4) and busulfan (4,800 μM^*^min) reported a maximum tolerated dose (MTD) of 9 Gy. NRM was 29%, relapse-free survival (RFS) was 43% and OS was 50% (319). A Phase I trial combining dose-escalated TMI from 12 to 18 Gy (3 Gy/day) with fludarabine (25 mg/m^2^ on days −9 to −7) and cyclophosphamide (60 mg/m^2^ on days −8 and −7), established 15 Gy as the MTD (320). Other groups are evaluating larger fraction sizes of up to 5 Gy in ongoing trials (32, 329, 330).

#### TMI or TMLI Added to Reduced-Intensity Conditioning Regimens

Reduced-intensity conditioning (RIC) regimens were developed for patients who cannot tolerate standard myeloablative regimens (331) and for paediatric patients where there are concerns regarding feasibility of myeloablative conditioning. These regimens are better tolerated and less cytotoxic but can be associated with a significant increase in relapse rates and a decrease in OS (332). Adding TMI/TMLI may achieve myeloablative medullary radiotherapy doses while not increasing risks for OAR. Rosenthal et al. successfully added 12 Gy TMLI (in eight fractions on days −7 to −4) (Figure 4B) to an RIC regimen of fludarabine (25 mg/m^2^/day on days −7 to −4) and melphalan (140 mg/m^2^ on day −2) in 61 patients (309, 321). Two-year OS was 54%, EFS was 49% and NRM was 30%. A successor Phase I trial of dose-escalated TMLI is ongoing, with a modified schedule of TMLI 12–20 Gy (days −9 to −5), fludarabine (30 mg/m^2^/day on days −4 to −2) and melphalan (100 mg/m^2^ on day −2). Welliver et al. are conducting an ongoing trial evaluating TMI and cyclophosphamide in patients who were unable to undergo myeloablative TBI (322).

#### TMI or TMLI Combined With GvHD Reduction Strategies

Strategies to reduce GvHD include the use of post-transplant cyclophosphamide (PTCy) (333, 334) and regulatory T cell/conventional T cell (T_reg_/T_con_) adoptive immunotherapy (325, 335). These regimens can also reduce graft vs. leukaemia effects. TMLI has been added to counterbalance this. In a Phase I trial, 29 patients with high-risk AML, ALL or myelodysplastic syndrome (MDS) received TMLI (12–20 Gy on days −7 to −3) combined with a regimen of fludarabine (25 mg/m^2^/day on days −7 to −4), cyclophosphamide (14.5 mg/kg/day on days −7 and −6), and PTCy (50 mg/kg on days +3 and +4), and reported a MTD for TMLI of 20 Gy (323). At 1 year, the cumulative incidence rate of relapse/progression was 24% and OS was 83%. Cumulative incidence of chronic GvHD was 25%. Day 100 and 1-year NRM rates were 4 and 9%, respectively (324). A Phase II trial is ongoing.

A recent Phase II trial of 50 patients with high-risk AML used T_reg_/T_con_ adoptive immunotherapy combined with myeloablative TMLI in patients >50 years (13.5 Gy to the bone marrow and 11.7 Gy to the lymph nodes in eight fractions) or TBI in patients ≤50 years (13.5 Gy in nine fractions or an 8 Gy single fraction) plus thiotepa, fludarabine, and cyclophosphamide. Moderate/severe chronic GvHD occurred in only one patient, NRM occurred in 10 patients, and only two patients relapsed. With a median follow-up of 29 months, the probability of moderate-to-severe chronic GvHD-free, relapse-free survival was 75% (325).

#### TMI or TMLI in Patients in First Remission as a Possible Alternative to TBI

TMI and TMLI are under investigation for patients in remission who normally would be eligible for standard TBI regimens (307). A pilot trial of TMLI of 20 Gy and PTCy reported a 2 year OS 86.7%, RFS of 83.3%, chronic GVHD incidence of 35% (moderate to severe 7%) and NRM of 0%, which compares favourably to the historical TBI experience (307). Other centres are evaluating IMRT-based organ sparing TBI in this population (127, 292, 336, 337).

### Long-Term Toxicities With TMI and TMLI

Long-term toxicities were recently reported in 142 patients receiving TMI (129, 305). The median dose was 14 Gy (range 10–19 Gy). One patient developed radiation pneumonitis (0.7%). Mean lung dose ≤8 vs. >8 Gy was predictive of significantly lower rates of both respiratory infection and IP at 2 years (21 vs. 32%, respectively, *p* = 0.01). The incidence of radiation-induced renal toxicity was 0%, hypothyroidism was 6% and cataract formation was 7%. The low incidence of toxicities compared with conventional TBI and the successful engraftment rates also suggest that higher dose rates with TMI do not significantly contribute to the incidence of marrow or organ toxicities.

### Extramedullary Relapses After TMI and TMLI

In a study assessing the incidence of extramedullary recurrences in 101 patients undergoing allogeneic HSCT following conditioning with TMLI, 13 patients developed extramedullary relapses at 19 sites. The site of relapse was not dose dependent, and the risk of extramedullary relapse observed was comparable to that previously reported with standard TBI, suggesting that TMLI did not increase the risk of relapse in non-target regions (338). This possibly indicates that the main added value of radiotherapy to conditioning before HSCT lies in its immunosuppressive ability and the eradication of leukaemic deposits in bone marrow, lymphatic volumes and sanctuary sites, and not so much in depleting extramedullary or circulating leukaemic cell volumes. The lower integral dose given over the entire body during TMI/TMLI may still function in eradicating small numbers of extramedullary or circulating leukaemic cells (339). Therefore, TMI/TMLI delivery techniques should not be withheld based on concerns of dose heterogeneity to extramedullary/extralymphoid sites.

## Conclusions and Future Directions

Myeloablative fractionated TBI delivered together with chemotherapy remains the standard for conditioning prior to HSCT in paediatric patients with high-risk or relapsed/refractory ALL. Since its introduction, TBI has undergone developments to decrease the risks of late sequelae. Still, survivors typically develop serious late effects and efforts to improve the balance between outcomes and toxicity need to continue. While TBI performance between different radiotherapy centres is heterogeneous, with many centres not changing practises for a long time, new techniques may have the potential to mitigate adverse effects while preserving efficacy. To properly evaluate real-world data, we need comparable TBI schedules, uniform specifications, and comprehensive standardised reporting of all relevant parameters. Cooperation between treatment centres and research groups can support new insights, implementation of new techniques and research regarding the potential to reduce the need for TBI, lower TBI doses, or decrease radiotherapy treatment volumes within the body. Future studies must identify whether highly conformal TBI or TMI/TMLI techniques offer equal disease outcomes while reducing toxicity.

## Author Contributions

All authors fulfil the criteria for authorship, wrote significant sections of this review, and reviewed and approved the entire manuscript.

## Conflict of Interest

The authors declare that the research was conducted in the absence of any commercial or financial relationships that could be construed as a potential conflict of interest.

## Publisher's Note

All claims expressed in this article are solely those of the authors and do not necessarily represent those of their affiliated organizations, or those of the publisher, the editors and the reviewers. Any product that may be evaluated in this article, or claim that may be made by its manufacturer, is not guaranteed or endorsed by the publisher.
